# Errata

**Published:** 2014

**Errata 1**

The article **“An optimal painless treatment for early hemorrhoids; our experience in Government Medical College and Hospital”**, authors R Singal, S Gupta, AK Dalal, U Dalal, AK Attri, was published in Journal of Medicine and Life, vol. VI, issue 3, pp. 302-306, 2013 in the “Case Presentation” category.

The above-mentioned article should be included in the “Original Article” category and the change should be made at all the levels, starting with the journal’s web page – www.medandlife.ro and ending with the databases the journal is indexed in (PubMed, Index Copernicus, EBSCO Publishing, ProQuest).

**Errata 2**

The article **“Comparative study on Complementary and Alternative Medicine (CAM) use by physicians in Romania and Hungary”**, authors Jupaneant O, Hegyi G, Tudor A, Purcarea VL, Dragan S, was published in Journal of Medicine and Life, vol. VII, issue 4, 2014, in the “Case Presentation” category. After its publication, the authors decided to withdraw the article in order to publish it in another journal.

As a result, it was decided to replace the above-mentioned article with the article **“Optical Coherence Tomography versus Visual Evoked Potentials in detecting subclinical visual impairment in multiple sclerosis”**, author Monica Grecescu.

**Errata 3**

The article **“Endo-periodontal lesion – endodontic approach”**, authors Jivoinovici R, Suciu I, Dimitriu B, Perlea P, Bartok R, Malita M, Ionescu C, was not published in Journal of Medicine and Life, vol. VII, issue 4, 2014, in the “Case Presentation” category, in the first published lot of the journal, due to the fact that another article, **“Comparative study on Complementary and Alternative Medicine (CAM) use by physicians in Romania and Hungary”**, was published and withdrawn afterwards.

As a result, it was decided that the article **“Endo-periodontal lesion – endodontic approach”** will be included in the final version of the journal.

**The publishers regret any inconvenience caused by these errors.**

